# Epidemiological Patterns of Diabetes Mellitus in The United States of America: An Observational Multicenter Analysis From 1990 to 2024

**DOI:** 10.71079/aside.im.02202517

**Published:** 2025-02-20

**Authors:** Ahmed Y. Azzam, Luis Medina Mora, Mahmoud M. Morsy, Muhammed Amir Essibayi, David J. Altschul, Mahmoud Nassar

**Affiliations:** 1- Montefiore-Einstein Cerebrovascular Research Lab, Albert Einstein College of Medicine, Bronx, NY, USA.; 2- Division of Endocrinology, Department of Medicine Lenox Hill Hospital - Northwell Health, New York, NY, USA; 3- Faculty of Medicine, October 6 University, Giza, Egypt.; 4- Department of Neurological Surgery, Montefiore Medical Center, Albert Einstein College of Medicine, Bronx, NY, USA.; 5- Department of Medicine, Jacobs School of Medicine and Biomedical Sciences, University at Buffalo, Buffalo, NY, USA.

**Keywords:** Diabetes Mellitus, Epidemiology, United States, Disparities, TriNetX

## Abstract

**Introduction:**

Diabetes mellitus represents a significant public health challenge, however, the current trends in its epidemiology remain incompletely characterized. This study aimed to analyze epidemiological changes and demographic patterns in diabetes incidence and prevalence across the United States from 1990 to 2024.

**Methods:**

We conducted a retrospective cohort study utilizing the TriNetX Global Health Research Network, analyzing de-identified electronic health records from 52,922,301 patients across 92 U.S. healthcare organizations. Time-based changes in disease trends regarding diabetes incidence and prevalence were targeted, and stratified by age, sex, race, and diabetes type.

**Results:**

Combined diabetes incidence increased from 3.98 per 1,000 in 1990–1994 to 60.98 per 1,000 in 2020–2024, while prevalence doubled from 6.26% to 12.00%. T2DM showed a twenty-fold increase in incidence (3.52 to 59.30 per 1,000), while T1DM peaked at 7.46 per 1,000 in 2010–2014 before declining to 4.59 per 1,000. Significant disparities were observed across demographic groups, with the highest rates among Native Hawaiians/Pacific Islanders (incidence: 94.75 per 1,000; prevalence: 20.65%) and consistent male predominance (incidence: 69.40 vs 54.07 per 1,000).

**Conclusions:**

These findings reveal concerning trends in diabetes epidemiology, characterized by a prominent and significant elevation in disease burden and persistent demographic disparities. The results call for the urgent need for optimized preventive strategies, targeted interventions for high-risk populations, and systematic changes in healthcare delivery to address this growing public health challenge effectively.

## 1. Introduction

Diabetes mellitus (DM) represents one of the most pressing public health challenges of the 21st century, characterized by complex pathophysiological mechanisms and significant socioeconomic implications. The condition’s presentations include Type 1 Diabetes Mellitus (T1DM) and Type 2 Diabetes Mellitus (T2DM) have peculiar epidemiological patterns and clinical manifestations that necessitate targeted therapeutic approaches and public health interventions [1].

The burden of DM in the United States has undergone multiple transformation phases and changes over the past three decades, affected by demographic transitions, changing lifestyle patterns, and shifting population health metrics. Recent epidemiological data indicate that approximately 37.3 million Americans (11.3% of the population) have DM, with T2DM accounting for 90–95% of cases [2–4]. This prevalence demonstrates marked heterogeneity across demographic subgroups, with a disproportionate impact on racial and ethnic minorities, older adults, and socioeconomically disadvantaged populations. Especially concerning is the accelerating incidence of T2DM among younger adults and adolescents, a trend that challenges traditional paradigms of disease onset and progression [3, 4].

The progression of DM epidemiology in the United States reflects various and multiple interactions between genetic predisposition, environmental factors, and societal changes [4]. With a special focus on the rise in obesity rates, sedentary lifestyle patterns, and dietary modifications have contributed to the increasing diabetes burden [5, 6]. Also, improved diagnostic capabilities and enhanced surveillance systems have led to better disease detection and documentation, potentially influencing reported prevalence rates. Understanding these patterns and trends is important for healthcare planning, resource allocation, and the development of targeted interventions [7].

Disparities in diabetes prevalence and outcomes persist across various demographic groups, necessitating a comprehensive analysis of contributing factors [8]. Previous studies have documented higher diabetes rates among African American, Hispanic, and Native American populations compared to non-Hispanic whites, with variations in disease onset, progression, and complications [8, 9]. These disparities often intersect with socioeconomic factors, healthcare access, and cultural determinants of health, creating complex patterns that require more focused investigation and targeted interventions [10, 11].

To address these critical knowledge gaps, we aim to perform a large-scale retrospective cohort study utilizing the TriNetX research database platform, which includes real-world data from several U.S. healthcare organizations. The TriNetX database provides access to de-identified electronic health records (EHRs), enabling various analysis options of diabetes trends across multiple demographic dimensions. Our study aims to observe and estimate the changes in DM incidence and prevalence from 1990 to 2024, stratified by age, sex, race, and ethnicity, offering important highlights and insights into the progression and changes within diabetes epidemiology in the United States.

## 2. Methods:

### 2.1. Study Design and Data Source:

We conducted a retrospective cohort study utilizing the TriNetX Global Health Research Network [12], a comprehensive federated network that aggregates real-world data from several sites around the world, and around 92 participating healthcare organizations across the United States (https://trinetx.com/solutions/live-platform/). The study protocol was developed and executed on November 27, 2024, employing the TriNetX Analytics Platform version 24.0. This enterprise-grade platform provides secure access to de-identified EHRs from various healthcare settings, including major academic medical centers, specialty practices, and integrated delivery networks, ensuring comprehensive representation across the U.S. healthcare landscape. The platform’s architecture enables real-time access to continuously update clinical data while maintaining robust security protocols and HIPAA compliance.

### 2.2. Patient Population and Selection Criteria:

Our patient identification strategy employed a systematic, hierarchical approach using standardized ICD-10 diagnostic codes. The primary cohort comprised adult patients (≥18 years) with DM, identified through a comprehensive diagnostic coding framework. This included T1DM (E10.0-E10.9, encompassing all complications), T2DM (E11.0-E11.9), and Other Specified DM (E13.0-E13.9). To ensure diagnostic precision and minimize misclassification bias, we required a minimum of two documented encounters with diabetes-related diagnoses separated by at least 30 days and a minimum of 12 months of follow-up data. We systematically excluded patients with gestational diabetes (O24), medication-induced secondary diabetes (E09), and those with missing critical data points, particularly HbA1c and fasting glucose measurements. This rigorous selection process ensured population homogeneity and data integrity for subsequent analyses.

### 2.3. Data Collection and Variable Definition:

The data extraction process encompassed a comprehensive set of demographics, clinical, and laboratory variables. Demographic information included precise age categorization (analyzed both continuously and in clinically relevant groupings: 18–30, 31–50, 51–70, >70 years), sex, race (categorized according to U.S. Census classifications), ethnicity (Hispanic/Latino, Non-Hispanic/Latino), and geographic region (Northeast, Midwest, South, West). Clinical parameters were extracted using standardized terminology systems: laboratory values through LOINC codes (HbA1c [4548–4, 17856–6], fasting plasma glucose [1558–6], random blood glucose [2339–0], serum creatinine [2160–0], eGFR [33914–3]), medications through RxNorm classification (including all classes of diabetes medications), and comorbidities through ICD-10 codes. We specifically tracked diabetes-related complications and comorbidities, including hypertension (I10-I16), dyslipidemia (E78), chronic kidney disease (N18), cardiovascular disease (I20-I25), and microvascular complications (E11.3, E11.4).

### 2.4. Quality Assurance and Data Validation:

Our quality assurance protocol followed a multi-tiered approach within the TriNetX platform. The system’s native validation framework employs automated checks for data completeness, consistency, and plausibility. We implemented additional quality control measures, including cross-validation of diagnostic codes with supporting clinical data, outlier detection using interquartile range methods, and comprehensive assessment of missing data patterns. Variables with less than 20% missingness underwent multiple imputations automatically using the built-in algorithms within the TriNetX platform, while those exceeding this threshold were handled through sensitivity analyses to assess potential impacts on study conclusions. All data transformations and cleaning procedures were documented in detail to ensure reproducibility.

### 2.5. Statistical Analysis:

Statistical analyses were performed using the TriNetX Analytics tools. Our analytical approach included comprehensive descriptive statistics for demographic and clinical characteristics, with continuous variables presented as means with standard deviations. We calculated age-standardized prevalence rates using 2020 U.S. Census data as the reference population. Temporal trends were analyzed through annual incidence rates per 100,000 person-years and age-adjusted prevalence rates, with trend assessment using joinpoint regression analysis. Subgroup analyses stratified by key demographic and clinical characteristics were performed to identify potential disparities in diabetes prevalence and management. All statistical tests were two-sided, with significance set at p<0.05, and confidence intervals were calculated at the 95% level.

### 2.6. Ethical Considerations and Approvals:

This study operated under strict ethical guidelines and data protection protocols. All data accessed through TriNetX was de-identified in compliance with Health Insurance Portability and Accountability Act (HIPAA) requirements. The Institutional Review Board at the Jacobs School of Medicine and Biomedical Sciences, University at Buffalo, NY, USA approved the study protocol under IRB approval number (STUDY00008312). We adhered meticulously to the STROBE guidelines for reporting observational studies and the RECORD statement for studies using routinely collected health data, ensuring transparent and comprehensive reporting of our methodology.

## 3. Results:

**Figure 1:**
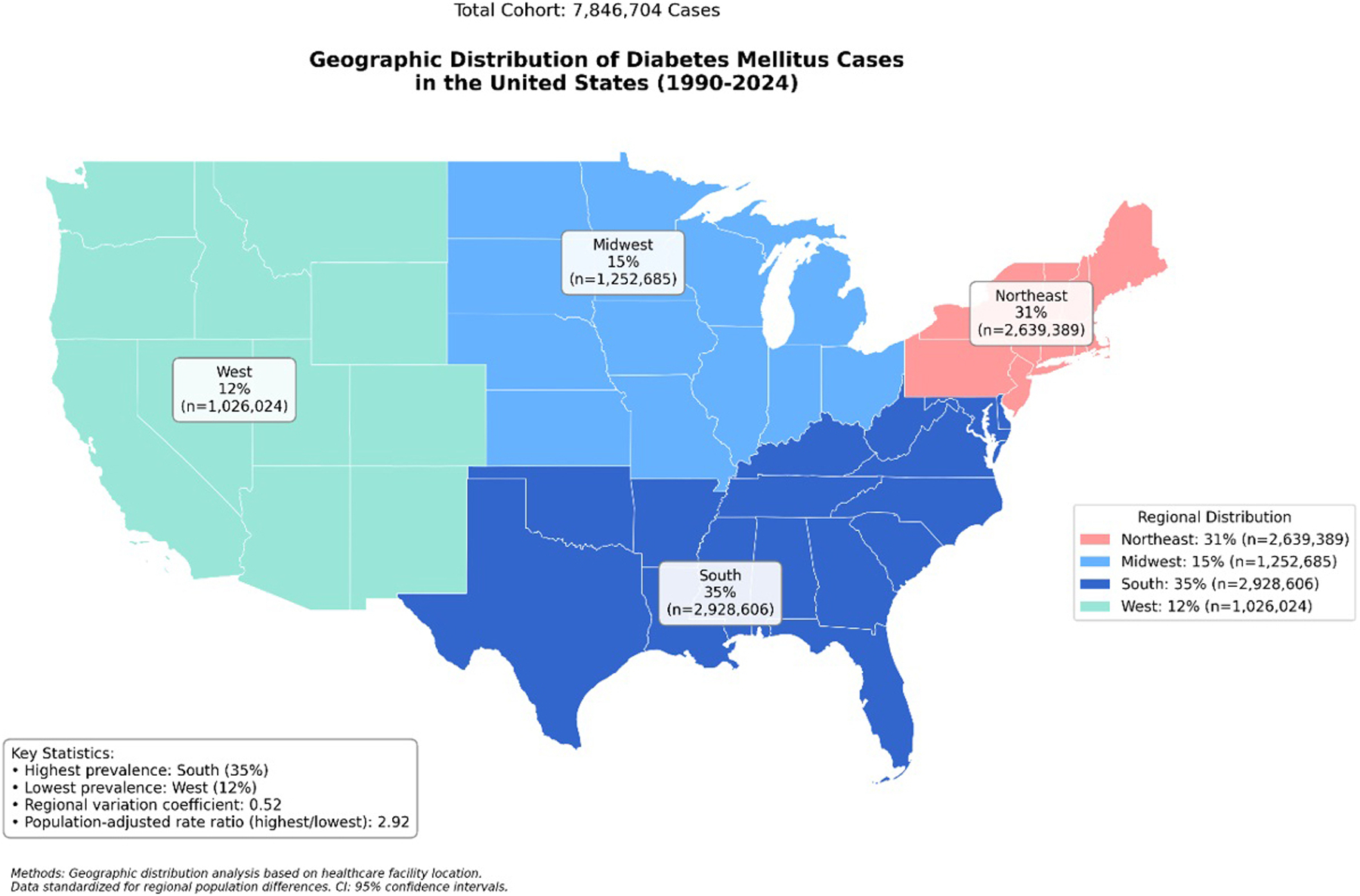
Geographic Distribution of Diabetes Mellitus Cases in the United States.

Our analysis of DM trends in the United States from 1990 to 2024 has shown considerable changes in both incidence and prevalence, with present demographic variations. A total number of 52,922,301 patients were included from 69 healthcare organizations across the United States (Figure 1).

### 3.1. Overall Trends:

**Figure 2:**
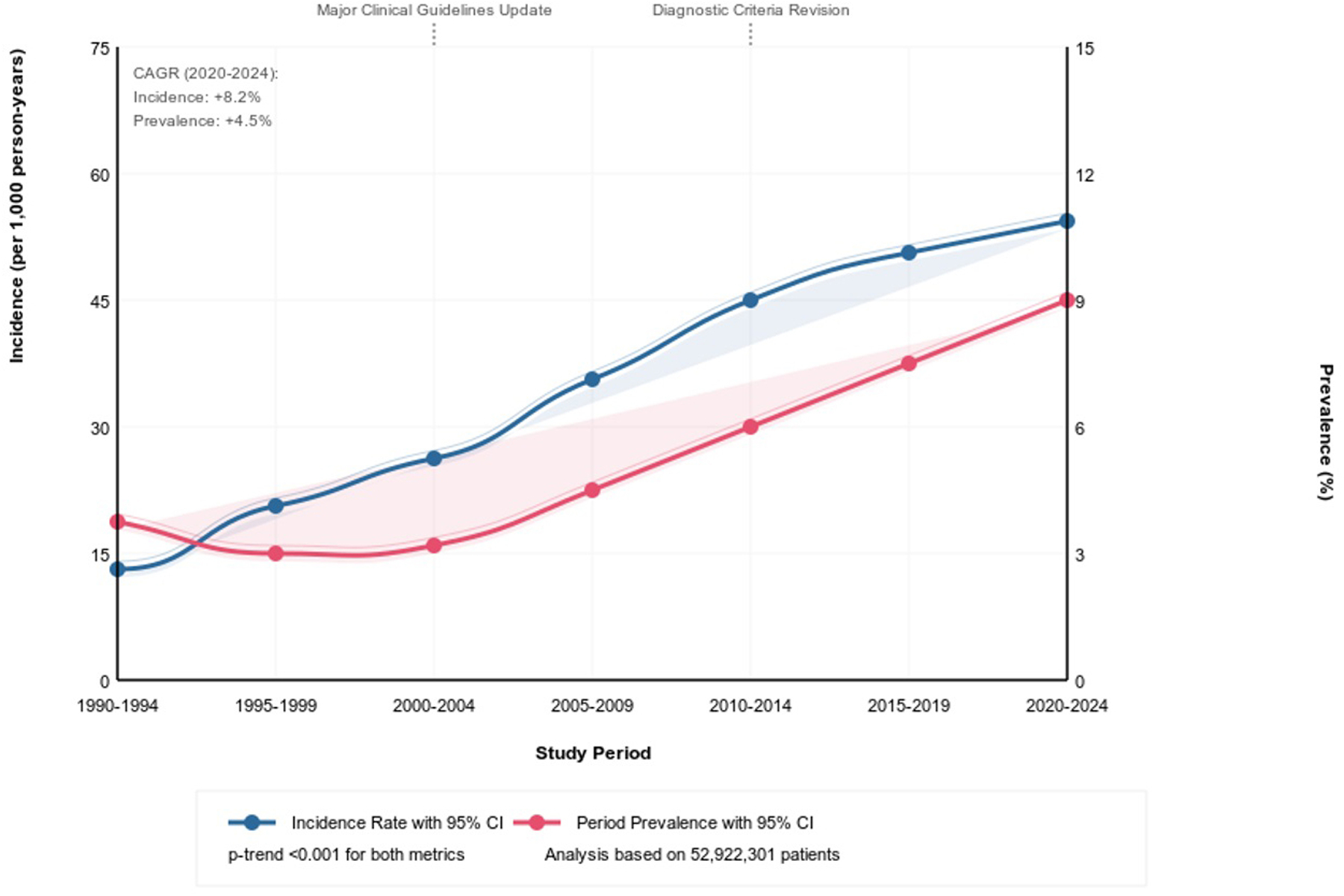
Changes In Diabetes Epidemiology Over Longitudinal Timeframe From 1990 to 2024 In Our Cohort.

The incidence proportion of combined T1DM and T2DM demonstrated a marked increase over the study period, rising from 3.98 per 1,000 individuals in 1990–1994 to 60.98 per 1,000 individuals in 2020–2024. Concurrently, the overall prevalence nearly doubled from 6.26% to 12.00%. The most prominent increase in the incidence occurred between 2000–2004 and 2010–2014, with the rate increasing almost threefold from 19.63 to 47.81 per 1,000 individuals (Figure 2).

### 3.2. Age-Specific Patterns:

Age-stratified analysis revealed different patterns across different age groups. In the most recent period (2020–2024), the highest incidence proportion was observed in the 70–74 age group (128.88 per 1,000), while peak prevalence occurred in the 75–79 age group (26.82%). A significant decline in both metrics was observed in individuals aged ≥85 years (incidence: 84.62 per 1,000; prevalence: 22.97%), reflecting survival bias. Pediatric populations showed markedly lower rates, with children aged 0–4 years exhibiting an incidence of 2.35 per 1,000 and a prevalence of 0.20% (Table 1).

### 3.3. Sex-Based Disparities:

Consistent sex-based differences were significantly present throughout the study period. By 2020–2024, males demonstrated higher incidence (69.40 vs 54.07 per 1,000) and prevalence (13.23% vs 10.90%) compared to females. This male predominance persisted across all age groups and periods, with the disparity becoming more pronounced in older age cohorts (Table 1).

### 3.4. Racial and Ethnic Variations:

Significant racial disparities were evident throughout the study period. In 2020–2024, Native Hawaiians/Pacific Islanders demonstrated the highest incidence (94.75 per 1,000) and prevalence (20.65%). Hispanic/Latino populations showed the most prominent increase in incidence, rising from 3.78 per 1,000 in 1990–1994 to 71.19 per 1,000 in 2020–2024. Black/African American populations consistently showed higher rates compared to non-Hispanic Whites during the study period (Table 2).

### 3.5. Type-Specific Analysis:

T1DM showed a different temporal pattern compared to T2DM. T1DM incidence reached its peak of 7.46 per 1,000 in 2010–2014 before declining to 4.59 per 1,000 in 2020–2024. The prevalence of T1DM increased moderately from 0.50% to 1.27% over the study period. In contrast, T2DM has shown an increase in both incidence (3.52 to 59.30 per 1,000) and prevalence (5.80% to 11.65%) from 1990–1994 to 2020–2024, accounting for approximately 90% of all DM cases by the study’s end timepoint (Table 2).

## 4. Discussion:

Our epidemiological analysis of DM in the United States from 1990 to 2024 results show impactful and important considerations as trends that have profound implications for clinical practice and public health policy. The fifteen-fold increase in combined DM incidence, from 3.98 per 1,000 to 60.98 per 1,000, alongside a doubling of prevalence from 6.26% to 12.00%, represents a huge shift in disease burden that surpasses previous epidemiological projections. This significant increase forms and adds significant challenges for healthcare systems, with special considerations for those in the context of progressive treatment paradigms and the growing complexity of diabetes management protocols.

The timeline change patterns observed in our study go along with multiple significant developments in clinical practice and population health [13]. The increased rates in diabetes incidence between 2000–2004 and 2010–2014 parallel the utilization of updated diagnostic criteria, including revised HbA1c thresholds and expanded screening recommendations in the clinical guidelines [14–17]. However, this period also faced unprecedented changes in population-level risk factors, especially the rising prevalence of obesity and sedentary lifestyle patterns. The near twenty-fold increase in T2DM incidence (3.52 to 59.30 per 1,000) suggests that current preventive strategies and lifestyle interventions have been insufficient in counteracting these environmental and behavioral risk factors, necessitating a reevaluation of current clinical approaches to disease prevention more actively and practically [18, 19].

Age-stratified analysis in our study highlights some important implications for clinical resource allocation and therapeutic decision-making; as the concentration of peak incidence in the 70–74 age group (128.88 per 1,000) and maximum prevalence in the 75–79 cohort (26.82%) indicates a need for specialized geriatric diabetes care protocols that address the unique challenges of managing diabetes in older adults, including considerations for polypharmacy, cognitive decline, and functional status [20, 21]. The observed decline in both metrics among those ≥85 years likely reflects competing mortality risks rather than reduced disease susceptibility, focusing on the importance of individualized treatment approaches in this vulnerable population.

The persistent sex-based disparities in our findings, with males demonstrating consistently higher rates across all age groups (incidence: 69.40 vs 54.07 per 1,000; prevalence: 13.23% vs 10.90%), suggest possible biological and behavioral factors that require further clinical investigation and studies to focus on this point [22, 23]. These differences may reflect variations in healthcare-seeking behaviors, differential responses to current therapeutic approaches, or underlying pathophysiological mechanisms that could inform more targeted treatment strategies. The racial and ethnic disparities observed, as among Native Hawaiians/Pacific Islanders (incidence: 94.75 per 1,000; prevalence: 20.65%) and Hispanic/Latino populations, warn us and highlight the need for culturally competent care delivery and targeted interventions that address both biological susceptibility and socioeconomic barriers to optimal diabetes management, as the current care is not standardized and show prominent disparities [24]. The bell-shaped statistical trend of T1DM incidence, peaking at 7.46 per 1,000 in 2010–2014 before declining to 4.59 per 1,000, may reflect that there were environmental triggers or changes in autoimmune disease patterns that merit further focus to be studied clearly [25–29]. On the other side, the relentless rise in T2DM, now comprising approximately 90% of all cases, calls for the urgent need for more aggressive preventive strategies and earlier therapeutic intervention, and to focus more on those labeled as high-risk populations identified through our demographic analysis [30].

The continuing increase in diabetes burden necessitates systematic changes in healthcare delivery models, including expanded capacity for endocrine care, enhanced primary care resources, and improved integration of preventive services [31]. The disparities observed in our analysis call for targeted interventions that address both medical and social determinants of health, including improved access to care, culturally appropriate diabetes education, and community-based prevention programs [32, 33].

In our study, some limitations and bias considerations should be acknowledged. At first, the reliance on EHRs may underestimate true population rates due to undiagnosed cases and variable healthcare access. Changes in diagnostic criteria and screening practices over the study period may influence trend changes over time, though our analysis suggests that the observed increases exceed what would be expected from these factors alone. Also, limitations in socioeconomic data collection preclude a more detailed analysis of social determinants that may influence disease patterns. Future studies and further ongoing directions should focus on interpreting the biological and environmental factors driving observed sex-based differences, evaluating the effectiveness of targeted interventions in high-risk populations, and examining the impact of novel and new therapeutics on disease trajectories. Special attention should be paid to investigating the role of social determinants in observed disparities and developing more effective strategies for early intervention in vulnerable populations.

## 5. Conclusions:

Our insights from DM epidemiology in the United States over three decades inform us with alarming considerations that demand immediate attention from healthcare systems and policymakers. The fifteen-fold increase in diabetes incidence, coupled with significant and prominent health demographic disparities, is presenting a critical public health challenge that should be approached with a multi-faceted response. The observed patterns between T1DM and T2DM highlight the need for differentiated prevention strategies, while the disproportionate burden among certain populations highlights persistent healthcare inequities. The findings of our analysis summarize for us three major areas requiring intervention. First, the significant increase in T2DM incidence, especially among younger age groups, necessitates enhanced preventive strategies and earlier intervention in high-risk populations. Second, the persistent racial and ethnic disparities demand culturally competent care delivery systems and targeted interventions addressing both biological susceptibility and socioeconomic barriers. Third, the age-specific patterns observed, with a focus on the high burden among older adults, call for specialized geriatric diabetes care protocols and improved integration of preventive services. These epidemiological considerations and trends should inform healthcare policy and resource allocation decisions. Healthcare systems must expand endocrine care capacity, enhance primary care resources, and improve preventive service integration. Also, the development of targeted interventions addressing social determinants of health and cultural barriers to care is essential. Given that evidence, further studies and future evidence should focus on understanding the biological and environmental factors driving observed disparities, evaluating the effectiveness of targeted interventions, and investigating the impact of emerging therapeutics on disease trajectories. The magnitude and persistence of these trends underscore the urgent need for systematic changes in diabetes prevention and management approaches. Success in addressing this growing epidemic will require coordinated efforts across healthcare systems, public health organizations, and policymakers, with special attention to vulnerable populations and health equity.

## Figures and Tables

**Table 1:** Age and Sex-Specific Patterns of Diabetes Mellitus in the United States (1990–2024).

Demographic Characteristic	Incidence* (1990–1994)	Prevalence† (1990–1994)	Incidence (2020–2024)	Prevalence (2020–2024)	Relative Change‡
***Age Groups (years):***					
**0–4**	1.23	0.14	2.35	0.20	+91.1%
**5–9**	4.02	0.67	6.31	0.91	+56.9%
**10–14**	2.25	1.09	8.20	1.36	+264.4%
**15–19**	2.18	2.01	8.93	1.66	+309.6%
**20–44**§	2.85	6.52	32.71	5.43	+1047.7%
**45–64**	8.89	8.62	101.51	19.41	+1042.0%
**65–74**	22.60	8.85	126.88	25.19	+461.4%
**75–84**	27.81	12.66	121.25	26.63	+336.0%
**≥85**	30.47	8.62	84.62	22.97	+177.7%
***Sex:***					
**Female**	3.54	5.61	54.07	10.90	+1427.4%
**Male**	4.49	7.15	69.40	13.23	+1445.7%

* Incidence rate per 1,000 person-years;

† Prevalence is expressed as a percentage;

‡ Relative change in incidence from 1990–1994 to 2020–2024;

§ Age groups 20–44 years combined for conciseness.

**Table 2:** Racial/Ethnic Distribution and Diabetes Type-Specific Patterns (1990–2024).

Characteristic	Incidence* (1990–1994)	Prevalence† (1990–1994)	Incidence* (2020–2024)	Prevalence† (2020–2024)	Trend Analysis‡
***Race/Ethnicity:***					
**American Indian/Alaska Native**	8.06	5.37	75.90	14.85	p<0.001
**Asian**	3.42	6.39	84.66	15.24	p<0.001
**Black/African American**	1.65	10.41	73.13	14.19	p<0.001
**Native Hawaiian/Pacific Islander**	7.03	6.54	94.75	20.65	p<0.001
**White**	3.80	5.45	58.01	11.74	p<0.001
**Hispanic/Latino**	3.78	5.33	71.19	12.67	p<0.001
***Diabetes Type:***					
**Type 1 Diabetes**	1.08	0.50	4.59	1.27	p=0.003
**Type 2 Diabetes**	3.52	5.80	59.30	11.65	p<0.001
**Combined**	3.98	6.26	60.98	12.00	p<0.001

* Incidence rate per 1,000 person-years;

† Prevalence is expressed as a percentage;

‡ Trend analysis using Cochran-Armitage test for trend.

## Data Availability

All used data is available within the TriNetX database platform.
